# Singapore KneE osTeoarthritis CoHort (SKETCH): protocol for a multi-centre prospective cohort study

**DOI:** 10.1186/s12891-023-06207-1

**Published:** 2023-02-07

**Authors:** Bryan Yijia Tan, Zack Zhong Sheng Goh, Chien Joo Lim, Michelle Jessica Pereira, Su-Yin Yang, Kelvin Guoping Tan, Alvin Chin Kwong Tan, Phyllis Liang, J. Haxby Abbott, Andrew M. Briggs, David J. Hunter, Soren T. Skou, Julian Thumboo, Josip Car

**Affiliations:** 1 Department of Orthopaedic Surgery, Woodlands Health, Singapore, Singapore; 2 Lee Kong Chian School of Medicine, Nanyang Technological University, Singapore, Singapore; 3 Department of Orthopaedic Surgery, Woodlands Health, National Healthcare Group, Singapore, Singapore; 4 Health Services Outcome Research, National Healthcare Group, Singapore, Singapore; 5 Psychology Service, Woodlands Health, Singapore, Singapore; 6 Department of Orthopaedic Surgery, Tan Tock Seng Hospital, Singapore, Singapore; 7 Department of Orthopaedic Surgery, Khoo Teck Puat Hospital, Singapore, Singapore; 8 Rehabilitation Research Institute of Singapore, Nanyang Technological University, Singapore, Singapore; 9 Centre for Musculoskeletal Outcomes Research, Department of Surgical Sciences, Dunedin School of Medicine, University of Otago, Dunedin, New Zealand; 10 Faculty of Health Sciences, Curtin School of Allied Health and Curtin enAble Institute, Curtin University, Perth, WA Australia; 11 Sydney Musculoskeletal Health, Kolling Institute, University of Sydney and Rheumatology Department, Royal North Shore Hospital, Sydney, Australia; 12 Research Unit for Musculoskeletal Function and Physiotherapy, Department of Sports Science and Clinical Biomechanics, University of Southern Denmark, Odense M, Denmark; 13 The Research Unit PROgrez, Department of Physiotherapy and Occupational Therapy, Næstved-Slagelse-Ringsted Hospitals, Slagelse, Denmark; 14 Department of Rheumatology and Immunology, Singapore General Hospital, Singapore, Singapore; 15 Duke-NUS Medical School, Singapore, Singapore

**Keywords:** Knee Osteoarthritis, Cohort, Outcomes, Asian, Prognosis, Healthcare Utilization, Psychosocial

## Abstract

**Background:**

Knee osteoarthritis (OA) is a leading cause of global disability. The understanding of the role of psychosocial factors in knee OA outcomes is still evolving particularly in an Asian context. The primary aim of this study is to explore psychosocial factors that prognosticate short and long-term clinical outcomes, productivity, and healthcare utilization in patients with knee OA. Secondary aims are to explore the mediation and directional relationships and the role it plays in predicting the discordance between self-reported measures (SRM), physical-performance measures (PPMs) and objective clinical parameters.

**Methods:**

A multi-centre prospective cohort study of community ambulant knee OA patients seeking treatment in the tertiary healthcare institutions in Singapore will be conducted. Patients with secondary arthritis, significant cognitive impairment, severe medical comorbidities or previous knee arthroplasty will be excluded. Primary clinical outcome measure is the Knee injury and OA Outcome Score-12 (KOOS-12). Baseline characteristics include sociodemographic status, arthritis status including symptom duration and radiographic severity, comorbidities and functional status through Charlson Comorbidities Index (CCI), Barthel Index (BI) and Parker Mobility Score (PMS). Psychosocial variables include social support, kinesiophobia, negative affect, self-efficacy, injustice, chronic illness shame and the built environment. Clinical outcomes include quality of life, physical performance, global assessment, satisfaction and physical activity levels. Productivity and healthcare utilization will be assessed by a modified OA Cost and Consequences Questionnaire (OCC-Q) and the Work Productivity and Activity Impairment Questionnaire (WPAI). Variables will be collected at baseline, 4, 12 months and yearly thereafter. Regression, mediation and structural equation modelling will be used for analysis.

**Discussion:**

Results will allow contextualization, identification, and phenotyping of the critical (and potentially modifiable) psychosocial parameters that predict positive clinical outcomes in the OA population to guide optimization and refinement of healthcare and community. This will facilitate: 1. identification of high-risk knee OA subpopulations that will likely experience poor outcomes and 2. formulation of targeted multidisciplinary comprehensive approaches to address these psychosocial factors to optimize non-surgical treatment care, maximize functional outcomes and create more value-based care model for knee OA.

**Ethics and dissemination:**

The study has been registered under clinicaltrials.gov registry (Identifier: NCT04942236).

## Background

Based on the global burden of disease study, musculoskeletal (MSK) disorders account for the largest cause of disability worldwide [1]. In particular, osteoarthritis (OA) alone is the 3^rd^ most rapidly rising condition associated with disability [2]. 528 million people worldwide suffer from symptomatic and activity-limiting OA affecting quality of life, sleep and mood [3]. Risk factors for knee OA can be divided into modifiable and non-modifiable risk factors [4]. Non-modifiable risk factors include age, gender, ethnicity, genetics and joint-level factors (joint alignment, previous injury). Modifiable risk factors include weight, occupation, sports and joint alignment [5]. These factors work together to influence the progression of disease and its accompanying functional limitations.

Treatment algorithms and disease prognosis for knee OA have traditionally been viewed through a biomedical lens [6]. However, this approach is now considered outdated and missing many important factors [7]. Patients who present with similar radiographic joint abnormalities could have significant differences in how they experience their OA pain with evidence showing a discordance between objective clinical and radiological markers and self-reported pain and disability [8]. Large scale prognostic studies have focused on predominantly biomedical factors but failed to predict pain and functional impairment with high levels of certainties [9]. Pain itself is a complicated phenomenon, manifested and potentially modified by a complex interplay of neuropathic, physiological, psychological, genetic, social and personal factors, each contributing to a multifactorial experience of pain and hence challenges in empirical measurement [10–13]. These intricacies of pain experiences call for a wider approach toward the understanding and treatment of pain by incorporating a psychosocial perspective.

Mounting evidence has emerged that posits that psychological and social outcomes caused by direct or indirect effects of OA pain can also worsen disease trajectory [14]. The pathway between pain and psychological symptoms can be reciprocal and potentially causational [15–17]. Prolonged pain experiences can lead to negative psychological outcomes, and vice versa. A recent longitudinal study found that greater perceived OA pain and dysfunction at baseline is found to be associated with a higher incidence of depression at follow up [18]. Systematic review-level evidence has also identified that anxiety and depression symptoms in patients with OA are associated with poor healthcare outcomes, including increased doctor visits, healthcare utilisation, medication prescription, poor surgical outcomes and post-surgical pain [14]. Luong et al. explored the social factors in OA, highlighting the overall paucity of research in this area with the research mainly focusing on social position (education, income and occupation) and proposed a framework to guide future research [19].

Despite all this, the understanding of the role of psychosocial factors in knee OA outcomes is still early and evolving with a paucity of research involving psychosocial outcomes in patients with knee OA particularly in an Asian context. Existing large prospective cohort studies such as the Multicentre OA Study (MOST), OA Initiative (OAI), and the Chinese Primary Knee OA Progression Cohort (CPKOPC) focused mostly on the biological rather than psychosocial factors [20, 21]. A cohort study to identify critical psychosocial factors that predicts the disease trajectory of this Asian patient population, in closer alignment with patient expressed needs to reduce inequities by focusing on care dimensions beyond the biomechanical lens [22] and guide the optimisation and refinement of existing services.

### Study aims

The primary aim is to identify critical psychosocial factors that prognosticate short and long-term clinical outcomes, productivity, and healthcare utilization in patients with knee OA.

The secondary aims are

1. To explore the mediation and directional relationship between the psychosocial factors with clinical outcome, productivity, and healthcare utilization outcome to support the development of a conceptual framework
2. To explore discordance between self-reported measures (SRM), physical-performance measures (PPMs), objective clinical parameters e.g. radiographic severity in knee OA and the extent to which psychosocial factors predicts the extent of discordance

## Methodology

### Study design

The Singapore KneE OA CoHort (SKETCH) study is a multi-centre, prospective cohort study. The reporting of the study will follow the Strengthening the Reporting of Observational Studies in Epidemiology (STROBE) guidelines [23]. The Prognostic Research Strategy (PROGRESS) framework was used to incorporate best practices for prognostic research as part of this study protocol [24].

### Study population and setting

In this first phase, patients with knee OA in Singapore seeking medical care from tertiary hospitals and institutions will be recruited. The recruitment locations of the study will primarily be recruited from the outpatient orthopaedic surgery and physiotherapy departments. A second phase is planned to be conducted among patients within primary care and the community.

### Recruitment

Patients who satisfy the inclusion criteria outlined in Table 1 will be recruited. Participants will be identified and recruited with a dual-pronged recruitment strategy. First, research coordinators will conduct a pre-screening of the patients list using the institutions’ appointment and/or medical record systems to identify eligible patients who will be attending the orthopaedic or phsyiotherapy clinic for the day. Second, study site collaborators and their clinical teams, guided by the study inclusion and exclusion criteria will support recruitment by identifying potentially appropriate patients and proactively referring them to the study team. Delegated research coordinators will approach the patients who meet at the outpatient clinics and explain the study’s objectives to the patients. The research coordinator will obtain consent from the patient and offer to administer the questionnaire with the patient at their preferred time and location, or for the patient to complete the questionnaires independently.

**Table 1:** Inclusion and exclusion criteria

Inclusion criteria	Exclusion criteria
NICE Clinical criteria for OA Knee *NICE Clinical criteria for OA knee: patient is 45 or over and has activity-related joint pain and has either no morning joint-related stiffness or morning stiffness that lasts no longer than 30 min*	Alternative diagnosis to knee OA e.g.: referred pain from hip/spine *If co-existing pathology is present, patient can still be recruited if the predominant symptoms are from the OA knee with documentation of the co-existing pathology*
Independent community ambulators with or without walking aids	Secondary arthritis e.g., inflammatory
	Inability to comply with study protocol e.g.: significant cognitive impairment
	Severe and unstable medical comorbidities significantly impairing activities of daily living and risk of serious adverse events as assessed by a medical specialist (e.g., New York Heart Association (NYHA) class 4 cardiac failure with significantly impaired effort tolerance, stroke with significant residual functional weakness, psychiatric disorders such as psychosis, terminal cancer with a less expectancy of less than 12 months)
	Previous knee arthroplasty (index knee or contralateral knee)
	Pregnant

### Outcome measurement

#### Baseline variables

The following baseline measures will be collected. Firstly, the sociodemographic status which will include education level, housing status, employment and income details based on the PROGRESS-Plus framework [25] which summarises a number of social stratification factors that potentially impact health opportunities. Secondly, the arthritis status including the symptom duration and the radiographic severity of knee OA based on the Kellgren-Lawrence Scale [26]. Thirdly, the co-morbidities and functional status including the Charlson comorbidity index (CCI) [27], Barthel Index for Activities of Daily Living (BI) [28] and Parker Mobility Score (PMS) [29].

#### Psychosocial variables

The selection of outcome measures was guided by a combination of (I) the themes identified from qualitative work by the study team on local knee OA patient population to identify the potential psychosocial factors that impact knee OA progression [30], (II) a review of international literature, and (III) consultation with local domain experts to ensure local contextualization. The themes identified from the qualitative study (i.e., social support; religion/spirituality; built environment; fear avoidance/kinesiophobia; negative affect, depression/anxiety; and self-image and identity, loss of face) informed the selection of the psychosocial outcomes including social support, religiosity, built environment, fear of movement, etc. [30].

Validated psychosocial questionnaires previously used in OA research (e.g., brief fear of movement (BFOM) [31] and Arthritis Self-Efficacy Scale (ASES) [32], Multidimensional Scale of Perceived Social Support (MSPSS) [33], Chronic pain acceptance questionnaire (CPAQ) will be used for this study. Other outcome measures that have not been administered or validated in the OA population, such as religiosity and built environment, have been adapted from similar questionnaires and refined further by the study team in conjunction with local subject domain experts, contextualizing the questions to the local context to ensure content validity. The set of outcome measures was pilot tested to check for comprehensibility of the items.

#### Clinical, productivity and healthcare utilization outcomes

Recommendations set out by the OA Research Society International (OARSI) for domains of interest pertaining to non-surgical management of OA were adopted (i.e., pain, physical function, activity level, global assessment, and quality of life, etc.) [34, 35] for the clinical outcomes. The primary outcome will be Knee OA and Outcomes Score-12 (KOOS-12) [36]. The KOOS score has previously been validated in Singapore [37]. Other domains include quality of life, global assessment and symptom satisfaction (Tables 2 and 3).

**Table 2:** Overview of outcome measures

Baseline Characteristics	Psychosocial Variables		Clinical, Productivity and Healthcare Utilization Outcomes	
**Sociodemographic**	**Depression and Anxiety**	Patient Health Questionnaire-4	**Knee Function**	Knee OA and Outcome Score (KOOS)
**Arthritis status**	**Pain catastrophizing/ fear of movement**	Brief fear of movement	**Quality of Life**	EQ-5D
**Comorbidities and Functional Status**	**Pain experience**	Pain intensity and interference PEG Scale	**Physical Performance**	Gait Speed Time-up-and-Go (TUG) Sit-to-stand (STS) Stair climb
	**Social support**	Multidimensional Scale of Perceived Social Support	**Physical Activity Level**	Step count UCLA activity level
	**Built Environment**^a^	Built environment questionnaire	**Dietary behaviour**	Dietary Questionnaire
	**Chronic Illness Shame**^a^	Chronic Illness Shame Scale	**Global assessment**	Global Perceived Effect
	**Religion and Spirituality**^a^	Religion questionnaire	**Symptom Satisfaction**	Patient Acceptable Symptom Scale
	**Injustice experience**^b^	Injustice Experience Questionnaire	**Cost, Productivity and Healthcare Utilization**	OA Cost and Consequences Questionnaire (including indirect costs and productivity)
	**Self-efficacy**^b^	Arthritis Self-Efficacy Scale	**OA Care Quality**^a^	OA Quality Indicator (OA-QI)

^a^To be administered in phase 1 of study

^b^To be administered in phase 2 of study

**Table 3:** Outcome measures

**1. Sociodemographic and clinical data**	
Sociodemographic data	Data of the participants’ age, gender, ethnicity, education level, housing type, marital status, living arrangement, occupation will be collected through self-report
Arthritis status	Clinical variables including arthritis profile, duration, mobility and radiographic severity (Kellegren-Lawrence), and will be collected through a combination of self-report and clinical data extraction
Comorbidity and Functional Status	Charlson comorbidity index [27], Barthel Index for Activities of Daily Living [28] and Parker Mobility Score [29]
**2. Knee function**	
** KOOS-12**	KOOS-12 is a 12-item assessment tool on the participants’ perception of their knee function in the domains of pain, function and daily living, and quality of life [36]. It is measured on a 5-point Likert scale from 0 to 4, with 4 questions in each domain and scored using summative scores in each domain
** Physical Performance**	4 physical performance activities encompass the key domains of functional activities 1. Gait speed 2. Timed up-and-go 3. 4-stair climb test 4. 30-s chair stand
**3. Quality of life**	
The EQ-5D-5L questionnaire, consisting of 5 domains (mobility, self-care, usual activities, pain/discomfort, and anxiety/depression) will be used to assess participant’s quality of life [43]. Each dimension contains 5 levels, from no problem to extreme problems. A 5-digit number which describes the patient’s health state will be generated based on the levels that participants selected for each item. A subsequent index can be computed based on the valuation of the number. A vertical visual analogue scale in the assessment tool also provides for a quantitative measure of participants’ perceived health status	
**4. Depression and anxiety**	
The Patient Health Questionnaire-4 (PHQ-4) is a 4-item questionnaire answered on a four-point Likert-scale to allow the measurement of core sign and symptoms of depression and anxiety [14]. Total score is determined by adding together the scores of each of the 4 items. Scores are rated as normal (0–2), mild (3–5), moderate (6–8), and severe (9–12)	
**5. Pain experience**	
Pain average (P), interference with Enjoyment of life (E), and interference with General activity (G) (PEG) will be used to assess the pain experience [44]. The PEG is a three-item measure derived from the Brief Pain Inventory (BPI) [11, 45] that measures average pain intensity (one item) and pain interference (two items). Patients rate their pain intensity on a numerical rating scale from 0 (no pain) to 10 (pain as bad as you can imagine) and pain interference with enjoyment of life and general activity from 0 (does not interfere) to 10 (completely interferes). A mean score from the three questions will be computed to derive the overall pain impact	
**6. Activity level**	
The UCLA activity score is a 10-point activity scale that assesses activity level based on 10 descriptive activity levels ranging from 1 (i.e., wholly inactive, dependent on others and cannot leave residence) to 10 (i.e., regular participation in impact sports) [38]	
**7. Dietary behaviours**	
A dietary related questionnaire will be used to survey the dietary habits of the participants over the past four months. Questions include the frequency of intake of deep-fried foods, fruits and vegetables, whole grains, and sugar, as well as frequency of over-eating, stress eating, and dietary intention. Responses are reviewed and domain scores will be derived where applicable	
**8. Global and satisfaction**	
** Global Assessment**	The Global Perceived Effect (GPE) scale assesses the patient’s perception of knee OA progression [46]. It is a single item measure with 7-point Likert scale
** Satisfaction with treatment**	Patient Acceptable Symptom Scale (PASS) consists of an item pertaining to the perceived satisfaction of knee function, with a binary yes/no response [47]
** Treatment failure**	Perceived treatment failure will be assessed by an item about the participant’s current condition relating to the failure of the current course of treatment, with a binary yes/no response
**9. Perceived quality of care**	
The OA Quality Indicator (OA-QI) questionnaire seeks to understand the self-reported standard and level of information that the patient was provided with by the healthcare organisation [48]	
**10. Costs**	
Data on the acute health services usage (surgical, medication, others), specialist/medical service usage, community services programme, aids and adaptation will be collected, and the cost on the services related to the knee will be retrieved from the hospital data and the patient-reported cost questionnaire based on the Work Productivity and Activity Impairment Questionnaire (WPAI) and OA Cost and Consequences Questionnaire (OCC-Q)	
**11. Psychosocial**	
** Fear of movement**	Brief Fear of Movement (BFOM) assesses the fear of movement that patient experience [49]. The questionnaire consists of six questions with a 4-point Likert scale ranging from 1 (strongly disagree) to 4 (strongly agree). A summative score of the six questions will be computed, with higher score indicating greater fear of movement
** Self-efficacy**	The Arthritis Self-Efficacy Scale (ASES) assesses how the participants’ confidence in performing certain daily tasks [50]. The summative score indicates the level of self-efficacy the participant has in managing their arthritis
** Pain acceptance**	Chronic Pain Acceptance Questionnaire – 2 (CPAQ-2) is a two-item questionnaire that explores participants’ acceptance of pain in their daily living [51]. Participants rate their response in a seven-point Likert scale range from never true (1) to always true (7)
** Injustice experience**	The Injustice Experience Questionnaire consists of 12 statements that assesses how participants’ perception of injustice in their health condition [52]. The responses will be recorded in a five-point Likert scale range from 0—not at all to 4—all the time, and a total score of all the questions will be computed
** Social support**	The Multidimensional Scale of Perceived Social Support (MSPSS) is used to measure patients’ perception of perceived social support [53]. There were 12 questions in the questionnaire, and each item is rated on a seven-point Likert-type response format (1—very strongly disagree; 7—very strongly agree). A total score is calculated by summing the results for all items. The possible score range is between 12 and 84, the higher the score the higher the perceived social support
** Chronic illness-related shame**	Chronic illness-related shame score (CISS) is a scale specifically focused on shame feelings derived from illness-related experiences [54]. The CISS composed of seven items measured on a five-point Likert scare range from 0 (Never True) to 4 (Always True)
** Religion and spirituality**	The religion and spirituality questionnaire is a 2-item assessment tool developed by the study team to assess the extent that religion and spirituality is a factor in helping the patient cope with their knee condition. The items are rated from 1 (very strongly agree) to 7 (very strongly disagree)
** Built environment**	The built environment questionnaire is a 5-item developed by the study team to assess the participant’s perceptions of accessibility of physical facilities and amenities around their place of residence. This was adapted from the International Physical Activity Questionnaire (IPAQ) environmental module [55]. The items are rated from 1 (very strongly agree) to 7 (very strongly disagree)

The choice of PPMs was based on the recommended OARSI performance test for functional testing in OA [35]. Gait speed timed up-and-go, 4-stair climb test and 30-s chair stand were chosen to encompass the key domains of functional activities from sit-to-stand, walking short distances, stair negotiation and ambulatory transitions. The UCLA activity score is a validated score that is recommended for use in patients with hip or knee OA [38].

The Panel on Cost-Effectiveness in Health and Medicine recommends the use of a societal perspective to ensure that potentially important indirect costs such as productivity and caregiver cost would not be omitted [39]. Cost and healthcare utilization data will be collected via hospital administrative databases and patient-reported questionnaires to estimate direct medical, direct non-medical and indirect costs. Indirect costs include health-related productivity loss due to knee OA [40] from absenteeism and presenteeism, measured with the Work Productivity and Activity Impairment Questionnaire (WPAI) [41]. The scope of the cost data collection was based on the validated OA Cost and Consequences Questionnaire (OCC-Q) [42] and adapted to the Singapore context to ensure that all relevant sources of cost were collected.

#### Outcome measures timepoints

Upon baseline outcome measure taking, participants will be followed up at 4 months, 12 months, and annually after that. To reduce respondent fatigue and keep questionnaire burden to the minimum, psychosocial variables will be administered in two phases (Table 2) with a core set of outcome measures collected at all time points. In addition, certain psychosocial variables eg. built environment, that are not anticipated to change significantly over time will only be taken at baseline (Table 4).

**Table 4:** Data collection at different time points

Measure	Baseline	4 month	12 month	24 month
**Baseline**				
Informed consent	✓			
Demographic	✓			
Arthritis status	✓			
Comorbidity	✓			
**Clinical and functional assessment**				
Knee function^a^	✓	✓	✓	✓
Quality of life^a^	✓	✓	✓	✓
Depression and anxiety^a^	✓	✓	✓	✓
Pain experience^a^	✓	✓	✓	✓
Activity level^a^	✓	✓	✓	✓
Dietary behaviours	✓	✓	✓	✓
Global assessment^a^		✓	✓	✓
Satisfaction with treatment^a^		✓	✓	✓
**Healthcare Utilization and Productivity Costs**^a^	✓	✓	✓	✓
**Quality of Care**	✓			
**Psychosocial**				
Fear of movement	✓	✓	✓	✓
Self-efficacy	✓	✓	✓	✓
Pain acceptance	✓	✓	✓	✓
Injustice experience	✓			
Social support	✓			
Chronic illness shame	✓			
Religion and spirituality	✓			
Built environment	✓			

^a^Core outcome measures

### Sample size calculation

Sample size was estimated using G*Power 3.1.9.2. With a small effect size of 0.05, 0.05 type I error, 0.95 power of study and 10 predictive factors to be included into the model, the study will need to recruit 262 subjects. The final estimated sample size will be 420 cases after considering 20% attrition rate at each time point [(262 × 0.2) × 3 + 262] up to 2 years. Recruitment will be continued through the entire period to maximize statistical power.

### Statistical analysis plan

Data will be explored, cleaned and analysedusing STATA version 16.0 [56]. Descriptive statistics will be used to describe the demographic and clinical characteristics of the participants. The distribution of the continuous data will be assessed using skewness, kurtosis as well as histogram. Continuous data will be presented as mean and standard deviation if the data is normally distributed, otherwise median and interquartile range. Categorical variables will be presented as frequency and percentage.

#### Prognostic factors that influence the rehabilitation outcomes

Linear regression modelling will be used to investigate the factors that influence the knee function of the patients. Simple linear regression will be used to explore the variables which significantly predict the outcome, and stepwise variable selection method will be used to build the multivariable models. Multicollinearity and interaction terms of the final model will be checked, and heteroscedasticity. The model fit will be checked and assessed using Hosmer-Leme show goodness of fit test. In secondary analysis, mixed effect generalized linear models will be used to explore the changes in outcomes across time as well as the factors predicting the outcome of interest. Mediation analyses will also be conducted to explore the potential mediating psychosocial factors between predictors with clinical outcomes. Structural equation modelling (SEM) cross lagged analysis, an analytical strategy used to describe reciprocal relationships or directional influences, between variables over time, will be used to estimate the directional effects of various psychosocial factors at different time points. Statistical significance will be denoted as *p* < 0.05.

#### Handling of missing data

The missing data percentage will be explored and addressed when appropriate. Logistic regression will be used to ascertain the missing data mechanism and the association between the missingness and baseline covariates. Where appropriate, multiple imputation with predictive mean matching (PMM) will be used to predict and impute the missing continuous data based on the observed baseline covariates at each follow-up. On the other hand, the imputation of binary outcomes will be done using the ‘miimputelogit’ package from STATA [57].

### Cohort retention strategies

Long questionnaires and repeated follow ups may also influence attrition rates. Appropriate retention strategies will be applied to support participant retention [58]. This study will take on a multi-pronged approach in retaining participants and reducing attrition rates across the follow up timepoints. First, the outcome assessor of the participants will be kept consistent such that each participant will only be liaising with one member of the study team where a rapport has been established. Second, the questionnaire administration will adopt a barrier reducing approach in which participants will have the choice to complete the questionnaire on their own via an online digital secure form, at a time and place of their convenience or through the use of a hardcopy form. Third, through the provision of a grocery voucher after completion of the questionnaire at every timepoint, it is hoped that participants will feel incentivized to stay in the study throughout the study period.

### Data collection and storage

The collected data will be monitored by the study team. Data quality measures include queries to identify outliers and missing data analysis will be performed. A unique identifier will be assigned to each participant after enrolment to ensure patient confidentiality. The data collected will be stored on the Research Electronic Data Capture (REDCAP) system, a secure web application widely used for clinical data management in research.

## Discussion and conclusion

The longitudinal data of psychosocial outcomes in patients with knee OA collected in this study will provide clinicians, researchers and policymakers with contextualised knowledge to inform more data-driven care and to create more value-based models of care for knee OA patients. Studying psychosocial factors in the context of the Singapore and the broader Asian culture is important because there are likely to be differences in the psychosocial characteristics and presentation in the local population compared to studies in other countries. A review by Sathiyamoorthy et al. of the cultural factors influencing OA care in Asian communities highlighted the key role that cultural factors play in the uptake of OA management practices among Asians and posited that greater awareness of these cultural factors may improve overall management of OA among Asian patients [59]. Much work is needed to contextualize, identify and phenotype the critical (and potentially modifiable) psychosocial parameters that aid positive clinical outcomes in the OA population to guide optimization and refinement of healthcare and community.

“Psychosocial phenotyping” is an increasingly powerful tool to optimize personalized self-management interventions for people with chronic diseases that have been shown to be effective in many other conditions [60]. Psychosocial phenotyping has already been done in local studies in other contexts and diseases such as cancer [61, 62], eye diseases [63], and frailty [64], where findings could help guide targeted interventions and inform policies. Psychosocial phenotyping through the identification of the key psychosocial predictive factors will allow for the practice of “Precision Medicine”. Within knee OA, efforts have been made to phenotype patients based on a host of clinical variables [65, 66] with some studies focusing specifically on psychological factors [67, 68]. Through understanding the directional and mediation relationships between the various psychosocial factors and its association with established self-reported measures (SRM), physical-performance measures (PPMs), objective clinical parameters such as radiographic severity, this will allow us to untangle the complex relationships between the various measures that have been used in knee OA and develop conceptual frameworks to support further research.

Results from this study can be used in several ways. Firstly, it can help raise awareness among clinicians who manage patients with knee OA to identify high risk knee OA subpopulations that will likely experience poor outcomes based on pre-existing factors. This would allow them to address these factors and potentially formulate targeted approaches to address these psychosocial factors to optimize non-surgical treatment care, maximize functional outcomes and reducing/delaying the need for expensive surgery. Secondly, the results will inform the development of holistic biopsychosocial integrated multidisciplinary care models that specifically targets high-risk population groups to intervene on prognostic psychosocial factors to optimize outcomes. Results can also be used to augment existing clinical practice guidelines and promote cross-disciplinary training and collaboration to further support integrated care models.

For this study, recruitment will primarily be centred around the tertiary hospitals as patients presenting to the hospital are more likely to be experiencing more symptomatic and debilitating knee OA with the corresponding associated psychosocial factors. This will allow for relationships between psychosocial factors and outcomes to be more clearly established through a well-defined hospital-based cohort. In the next phase upon completion of this study, there are plans for subsequent phases of the cohort study to be expanded further downstream to primary care and the community and to explore other psychosocial factors that emerge over time as part of the larger effort to develop a comprehensive biopsychosocial understanding of knee OA.

## Data Availability

Not applicable.

## Abbreviations

OA: Osteoarthritis
SRM: Self-reported measures
PPM: Physical-performance measures
KOOS-12: Knee injury and OA Outcome Score-12
CCI: Charlson Comorbidities Index
BI: Barthel Index
PMS: Parker Mobility Score
OCC-Q: OA Cost and Consequences Questionnaire
WPAI: Work Productivity and Activity Impairment Questionnaire
MSK: Musculoskeletal
STROBE: Strengthening the Reporting of Observational Studies in Epidemiology
PROGRESS: Prognostic Research Strategy
BFOM: Brief fear of movement
ASES: Arthritis Self-Efficacy Scale
MSPSS: Multidimensional Scale of Perceived Social Support
CPAQ: Chronic pain acceptance questionnaire
OARSI: Osteoarthritis Research Society International
TUG: Time-up-and-Go
STS: Sit-to-stand
OA-QI: Osteoarthritis Quality Indicator
PHQ-4: Patient Health Questionnaire-4
GPE: Global Perceived Effect
PASS: Patient Acceptable Symptom Scale
CISS: Chronic illness-related shame score
IPAQ: International Physical Activity Questionnaire
REDCAP: Research Electronic Data Capture
NYHA: New York Heart Association

## References

[1] Cross M, Smith E, Hoy D, Nolte S, Ackerman I, Fransen M (2014). The global burden of hip and knee osteoarthritis: estimates from the global burden of disease 2010 study. Ann Rheum Dis.

[2] March L, Cross M, Lo C, Arden N, Gates L, Leyland K, et al. Osteoarthritis: a serious disease. OARSI Org. 2016. https://oarsi.org/oarsi-white-paper-oa-serious-disease.

[3] Long H, Liu Q, Yin H, Wang K, Diao N, Zhang Y (2022). Prevalence trends of site-specific osteoarthritis from 1990 to 2019: findings from the global burden of disease study 2019. Arthritis Rheumatol.

[4] Bennell KL, Hunter DJ, Hinman RS (2012). Management of osteoarthritis of the knee. BMJ.

[5] Silverwood V, Blagojevic-Bucknall M, Jinks C, Jordan JL, Protheroe J, Jordan KP (2015). Current evidence on risk factors for knee osteoarthritis in older adults: a systematic review and meta-analysis. Osteoarthritis Cartilage.

[6] Leung YY, Pua YH, Thumboo J (2013). A Perspective on Osteoarthritis Research in Singapore. Proc Singap Healthc.

[7] Bartley EJ, Palit S, Staud R (2017). Predictors of osteoarthritis pain: the importance of resilience. Curr Rheumatol Rep.

[8] Finan PH, Buenaver LF, Bounds SC, Hussain S, Park RJ, Haque UJ (2013). Discordance between pain and radiographic severity in knee osteoarthritis: findings from quantitative sensory testing of central sensitization. Arthritis Rheum.

[9] Baumbach L, List M, Grønne DT, Skou ST, Roos EM, Baumbach L (2020). Individualized predictions of changes in knee pain, quality of life and walking speed following patient education and exercise therapy in patients with knee osteoarthritis - a prognostic model study. Osteoarthritis Cartilage.

[10] Gwilym SE, Pollard TCB, Carr AJ (2008). Understanding pain in osteoarthritis. J Bone Joint Surg Br.

[11] Diatchenko L, Nackley AG, Slade GD, Fillingim RB, Maixner W (2006). Idiopathic pain disorders–pathways of vulnerability. Pain.

[12] Baker SL, Kirsch I (1991). Cognitive mediators of pain perception and tolerance. J Pers Soc Psychol.

[13] Bates MS, Edwards WT, Anderson KO (1993). Ethnocultural influences on variation in chronic pain perception. Pain.

[14] Sharma A, Kudesia P, Shi Q, Gandhi R (2016). Anxiety and depression in patients with osteoarthritis: impact and management challenges. Open Access Rheumatol Res Rev.

[15] Kroenke K, Wu J, Bair MJ, Krebs EE, Damush TM, Tu W (2011). Reciprocal relationship between pain and depression: a 12-month longitudinal analysis in primary care. J Pain.

[16] James RJE, Walsh DA, Ferguson E (2018). General and disease-specific pain trajectories as predictors of social and political outcomes in arthritis and cancer. BMC Med.

[17] James RJE, Ferguson E (2020). The dynamic relationship between pain, depression and cognitive function in a sample of newly diagnosed arthritic adults: a cross-lagged panel model. Psychol Med.

[18] Zheng S, Tu L, Cicuttini F, Zhu Z, Han W, Antony B (2021). Depression in patients with knee osteoarthritis: risk factors and associations with joint symptoms. BMC Musculoskelet Disord.

[19] Luong MLN, Cleveland RJ, Nyrop KA, Callahan LF (2012). Social determinants and osteoarthritis outcomes. Aging Health.

[20] Segal NA, Nevitt MC, Gross KD, Hietpas J, Glass NA, Lewis CE, et al. The Multicenter Osteoarthritis Study (MOST): Opportunities for Rehabilitation Research. PM R. 2013;5(8):10.1016/j.pmrj.2013.04.014. 10.1016/j.pmrj.2013.04.014 PMC3867287 23953013

[21] Eckstein F, Wirth W, Nevitt MC (2012). Recent advances in osteoarthritis imaging—the Osteoarthritis Initiative. Nat Rev Rheumatol.

[22] Slater H, Jordan JE, O’Sullivan PB, Schütze R, Goucke R, Chua J, et al. ‘Listen to me, learn from me’: a priority setting partnership for shaping interdisciplinary pain training to strengthen chronic pain care. Pain. 2022;163(11):e1145–e1163. 10.1097/j.pain.0000000000002647 PMC9578532 35384928

[23] von Elm E, Altman DG, Egger M, Pocock SJ, Gøtzsche PC, Vandenbroucke JP (2007). Strengthening the Reporting of Observational Studies in Epidemiology (STROBE) statement: guidelines for reporting observational studies. BMJ.

[24] Hemingway H, Croft P, Perel P, Hayden JA, Abrams K, Timmis A (2013). Prognosis research strategy (PROGRESS) 1: a framework for researching clinical outcomes. BMJ.

[25] O’Neill J, Tabish H, Welch V, Petticrew M, Pottie K, Clarke M (2014). Applying an equity lens to interventions: using PROGRESS ensures consideration of socially stratifying factors to illuminate inequities in health. J Clin Epidemiol.

[26] Kellgren JH, Lawrence JS (1957). Radiological assessment of osteo-arthrosis. Ann Rheum Dis.

[27] Charlson ME, Pompei P, Ales KL, MacKenzie CR (1987). A new method of classifying prognostic comorbidity in longitudinal studies: development and validation. J Chronic Dis.

[28] Mahoney FI, Barthel DW (1965). Functional evaluation: the barthel index. Md State Med J.

[29] Parker MJ, Palmer CR (1993). A new mobility score for predicting mortality after hip fracture. J Bone Joint Surg Br.

[30] Yang SY, Woon EYS, Griva K, Tan BY. A Qualitative Study of Psychosocial Factors in Patients With Knee Osteoarthritis: Insights Learned From an Asian Population. Clin Orthop. 2022. 10.1097/CORR.0000000000002526. 10.1097/CORR.0000000000002526 PMC10097569 36580492

[31] Thoma LM, Wolf K, Harris L, Best T, Flanigan D, Schmitt L (2015). Understanding fear of movement and reinjury with the Tampa scale of kinesiophobia (TSK) in individuals with musculoskeletal knee pathology: a systematic review. Osteoarthritis Cartilage.

[32] Brand E, Nyland J, Henzman C, McGinnis M (2013). Arthritis self-efficacy scale scores in knee osteoarthritis: a systematic review and meta-analysis comparing arthritis self-management education with or without exercise. J Orthop Sports Phys Ther.

[33] Ilori T, Ladipo MM, Ogunbode AM, Obimakinde AM (2016). Knee osteoarthritis and perceived social support amongst patients in a family medicine clinic. South Afr Fam Pract.

[34] Bannuru RR, Osani MC, Vaysbrot EE, Arden NK, Bennell K, Bierma-Zeinstra SMA (2019). OARSI guidelines for the non-surgical management of knee, hip, and polyarticular osteoarthritis. Osteoarthritis Cartilage.

[35] Dobson F, Hinman RS, Roos EM, Abbott JH, Stratford P, Davis AM (2013). OARSI recommended performance-based tests to assess physical function in people diagnosed with hip or knee osteoarthritis. Osteoarthritis Cartilage.

[36] Gandek B, Roos EM, Franklin PD, Ware JE (2019). A 12-item short form of the Knee injury and Osteoarthritis Outcome Score (KOOS-12): tests of reliability, validity and responsiveness. Osteoarthritis Cartilage.

[37] Leung YY, Ma S, Noviani M, Wong SBS, Lee CM, Soh IAL (2018). Validation of screening questionnaires for evaluation of knee osteoarthritis prevalence in the general population of Singapore. Int J Rheum Dis.

[38] Terwee CB, Bouwmeester W, van Elsland SL, de Vet HC, Dekker J (2011). Instruments to assess physical activity in patients with osteoarthritis of the hip or knee: a systematic review of measurement properties. Osteoarthritis Cartilage.

[39] Sanders GD, Neumann PJ, Basu A, Brock DW, Feeny D, Krahn M (2016). Recommendations for conduct, methodological practices, and reporting of cost-effectiveness analyses: second panel on cost-effectiveness in health and medicine. JAMA.

[40] Rice DP (1967). Estimating the cost of illness. Am J Public Health Nations Health.

[41] Reilly MC, Zbrozek AS, Dukes EM (1993). The validity and reproducibility of a work productivity and activity impairment instrument. Pharmacoeconomics.

[42] Pinto D, Robertson MC, Hansen P, Abbott JH (2011). Good agreement between questionnaire and administrative databases for health care use and costs in patients with osteoarthritis. BMC Med Res Methodol.

[43] Luo N, Wang P, Thumboo J, Lim YW, Vrijhoef HJM (2014). Valuation of EQ-5D-3L health states in Singapore: modeling of time trade-off values for 80 empirically observed health states. Pharmacoeconomics.

[44] Krebs EE, Lorenz KA, Bair MJ, Damush TM, Wu J, Sutherland JM (2009). Development and initial validation of the PEG, a three-item scale assessing pain intensity and interference. J Gen Intern Med.

[45] Poquet N, Lin C (2016). The Brief Pain Inventory (BPI). J Physiother.

[46] Scott W, McCracken LM (2015). Patients’ impression of change following treatment for chronic pain: global, specific, a single dimension, or many?. J Pain.

[47] Dougados M, Moore A, Yu S, Gitton X (2007). Evaluation of the patient acceptable symptom state in a pooled analysis of two multicentre, randomised, double-blind, placebo-controlled studies evaluating lumiracoxib and celecoxib in patients with osteoarthritis. Arthritis Res Ther.

[48] Østerås N, Garratt A, Grotle M, Natvig B, Kjeken I, Kvien TK (2013). Patient-reported quality of care for osteoarthritis: development and testing of the osteoarthritis quality indicator questionnaire. Arthritis Care Res.

[49] Shelby RA, Somers TJ, Keefe FJ, DeVellis BM, Patterson C, Renner JB (2012). Brief fear of movement scale for osteoarthritis. Arthritis Care Res.

[50] Brady TJ (2011). Measures of self-efficacy: Arthritis Self-Efficacy Scale (ASES), Arthritis Self-Efficacy Scale-8 Item (ASES-8), Children’s Arthritis Self-Efficacy Scale (CASE), Chronic Disease Self-Efficacy Scale (CDSES), Parent’s Arthritis Self-Efficacy Scale (PASE), and Rheumatoid Arthritis Self-Efficacy Scale (RASE). Arthritis Care Res.

[51] Vowles KE, Kruger ES, Bailey RW, Sowden G, Ashworth J, Hickman J (2020). Initial evaluation of the Chronic Pain Acceptance Questionnaire - 2. Eur J Pain Lond Engl.

[52] Yakobov E. Validation of the Injustice Experiences Questionnaire Adapted for Use with Patients with Severe Osteoarthritis of the Knee. J Arthritis. 2014 [cited 2022 Nov 25];03(02). Available from: http://www.omicsgroup.org/journals/validation-of-the-injustice-experiences-questionnaire-adapted-for-use-with-patients-with-severe-osteoarthritis-of-the-knee-2167-7921.1000130.php?aid=26697

[53] Zimet GD, Dahlem NW, Zimet SG, Farley GK (1988). The multidimensional scale of perceived social support. J Pers Assess.

[54] Trindade IA, Ferreira C, Pinto-Gouveia J (2017). Chronic illness-related shame: development of a new scale and novel approach for ibd patients’ depressive symptomatology. Clin Psychol Psychother.

[55] Alexander A, Bergman P, Hagströmer M, Sjöström M (2006). IPAQ environmental module; reliability testing. J Public Health.

[56] StataCorp. Stata Statistical Software: Release 16. College Station: StataCorp LLC; 2019.

[57] Little RJA (1988). Missing-data adjustments in large surveys. J Bus Econ Stat.

[58] Teague S, Youssef GJ, Macdonald JA, Sciberras E, Shatte A, Fuller-Tyszkiewicz M (2018). Retention strategies in longitudinal cohort studies: a systematic review and meta-analysis. BMC Med Res Methodol.

[59] Sathiyamoorthy T, Ali SA, Kloseck M (2018). Cultural factors influencing osteoarthritis care in asian communities: a review of the evidence. J Community Health.

[60] Kim MT, Radhakrishnan K, Heitkemper EM, Choi E, Burgermaster M (2021). Psychosocial phenotyping as a personalization strategy for chronic disease self-management interventions. Am J Transl Res.

[61] Liu J, Lam K, Mahendran R (2022). Psychosocial concerns predict longitudinal trajectories of distress in newly diagnosed cancer patients. Singapore Med J.

[62] Lam KFY, Lim HA, Kua EH, Griva K, Mahendran R (2018). Mindfulness and cancer patients’ emotional states: a latent profile analysis among newly diagnosed cancer patients. Mindfulness.

[63] Gupta P, Man REK, Fenwick EK, Aravindhan A, Gan AT, Thakur S (2020). Rationale and Methodology of The PopulatION HEalth and Eye Disease PRofile in Elderly Singaporeans Study [PIONEER]. Aging Dis.

[64] Teo N, Yeo PS, Gao Q, Nyunt MSZ, Foo JJ, Wee SL (2019). A bio-psycho-social approach for frailty amongst Singaporean Chinese community-dwelling older adults – evidence from the Singapore Longitudinal Aging Study. BMC Geriatr.

[65] Deveza LA, Melo L, Yamato TP, Mills K, Ravi V, Hunter DJ (2017). Knee osteoarthritis phenotypes and their relevance for outcomes: a systematic review. Osteoarthritis Cartilage.

[66] Deveza LA, Nelson AE, Loeser RF (2019). Phenotypes of osteoarthritis: current state and future implications. Clin Exp Rheumatol.

[67] Lentz TA, George SZ, Manickas-Hill O, Malay MR, O’Donnell J, Jayakumar P (2020). What general and pain-associated psychological distress phenotypes exist among patients with hip and knee osteoarthritis?. Clin Orthop.

[68] Pan F, Tian J, Cicuttini F, Jones G, Aitken D (2019). Differentiating knee pain phenotypes in older adults: a prospective cohort study. Rheumatol Oxf Engl.

